# Identification and Characterization of Biosecurity Breaches on Poultry Farms with a Recent History of Highly Pathogenic Avian Influenza Virus Infection Determined by Video Camera Monitoring in the Netherlands

**DOI:** 10.3390/pathogens14080751

**Published:** 2025-07-30

**Authors:** Armin R. W. Elbers, José L. Gonzales

**Affiliations:** Department of Epidemiology and Bioinformatics, Wageningen Bioveterinary Research, 8221 RA Lelystad, The Netherlands; jose.gonzales@wur.nl

**Keywords:** highly pathogenic avian influenza, biosecurity, anteroom, hand washing, hygiene lock, footwear, video monitoring, poultry

## Abstract

Biosecurity measures applied on poultry farms, with a recent history of highly pathogenic avian influenza virus infection, were monitored using 24 h/7 days-per-week video monitoring. Definition of biosecurity breaches were based on internationally acknowledged norms. Farms of four different production types (two broiler, two layer, two breeder broiler, and one duck farm) were selected. Observations of entry to and exit from the anteroom revealed a high degree of biosecurity breaches in six poultry farms and good biosecurity practices in one farm in strictly maintaining the separation between clean and potentially contaminated areas in the anteroom. Hand washing with soap and water and/or using disinfectant lotion was rarely observed at entry to the anteroom and was almost absent at exit. Egg transporters did not disinfect fork-lift wheels when entering the egg-storage room nor change or properly disinfect footwear. The egg-storage room was not cleaned and disinfected after egg transport by the farmer. Similarly, footwear and trolley wheels were not disinfected when introducing young broilers or ducklings to the poultry unit. Biosecurity breaches were observed when introducing bedding material in the duck farm. This study shows a need for an engaging awareness and training campaign for poultry farmers and their co-workers as well as for transporters to promote good biosecurity practices.

## 1. Introduction

In the last decade, Europe has suffered devastating highly pathogenic avian influenza (HPAI) epidemics, with thousands of outbreaks on poultry farms and an unprecedented number of dead wild birds [1]. In periods with enhanced risk of HPAI virus introduction into poultry farms (e.g., during the wild bird migration season), finding dead wild birds infected with HPAI virus (HPAIv) is a trigger to mandate housing all free-range poultry indoors in the Netherlands [2]. Despite the fact that direct contact between poultry and infected wild birds is prevented when poultry is housed indoors, many poultry farms became infected without conclusive evidence about the route of entry [2]. Several putative introduction pathways have been proposed like introduction of contaminated bedding material into poultry housing, unsafe drainage of rain-water from roof-tops of poultry buildings, transmission by insects and vermin (rats and mice), but also incidental breaches of biosecurity by the farmer or visitors [2].

Biosecurity is the first line of defence against introduction of pathogens. An effective biosecurity plan for poultry farms is based on two main concepts: exclusion and containment [3]. Exclusion or external biosecurity refers to preventive measures that reduce risks of introduction of infectious agents to non-infected flocks. Containment or internal biosecurity comprises measures to prevent transmission to different poultry units (physically separate rooms housing poultry) within the poultry farm when the pathogen is introduced into the farm. Effective biosecurity measures depend on the formation of a successful barrier between the farm and the outside environment. This sounds simple but, in practice, can be difficult to implement [4]. People, equipment, materials, and vehicles may frequently enter poultry farms. In addition, it is difficult to completely prevent the access of vermin to poultry houses. Examples of detailed biosecurity plans are available within the poultry industry [5]. It is of paramount importance that biosecurity measures are applied very strictly and consistently. It is important to have a clear separation between the possibly contaminated area of the premises outside the poultry house and the clean area of the inside of the anteroom and the poultry unit in which the poultry is housed [6,7]

Direct observation of how biosecurity measures are applied generates the most accurate data on possible biosecurity breaches, although the monitoring results may not represent perfectly the actual situation [8]. Monitoring hand hygiene compliance of health care workers using the direct, overt observation method has been accepted by experts as the gold standard [9]. A presumed weakness of direct observation is the possible occurrence of an undesired observer effect (Hawthorne effect), the phenomenon where individuals participating in research studies alter their behaviour because they are aware they are being observed [10,11]. Direct observation may improve compliance to biosecurity measures as a consequence of the awareness of being observed [12,13,14,15]. The use of human observers in direct observation has several drawbacks like being time-consuming and expensive, limitations with respect to inter-observer reliability, and differences in auditor skills [16]. Various electronic methods, including video monitoring, have been suggested as alternatives for human observers in order to limit a possible observer effect [16]. The use of video camera recording to investigate biosecurity breaches is known in human medicine, e.g., from studies with respect to hand hygiene compliance [15,17,18]. In the veterinary domain, Racicot et al. [4] were the first to provide a detailed report on biosecurity breaches on Canadian poultry farms observed by video monitoring. At the time, they used hidden cameras to observe application of biosecurity measures by farm workers. Despite potential limitations of using overt video camera monitoring, potential observer effects may be short-lived and may decline quickly over time [19].

The aim of our study was to identify and characterize biosecurity breaches of farmers, visitors, and transporters at entry and exit zones of farm buildings using 24 h/7 days-per-week video monitoring for 4 to 6 weeks on seven poultry farms of different production types with a recent history of HPAIv infection.

## 2. Materials and Methods

### 2.1. Study Population

The target population for this study consisted of 70 poultry farms infected one or more times in the last three years in the Netherlands with HPAIv of subtype H5Nx. For practical and budget limitations, we aimed to select a maximum of eight farms representing different poultry production types. To this end, out of the target population, a total of 27 farms including 9 laying hen farms (layer), 10 broiler farms (broiler), 6 meat duck farms (duck), and 2 broiler breeder farms (broiler) were randomly approached to ask for their willingness to participate. Farms with a free-range system with access to an uncovered outdoor range were excluded because, for these farms, effective types of biosecurity measures to reduce HPAIv risks differ from indoor farming systems due to the inherent exposure to the outside environment [20]. Poultry farms of different production types were approached because of a presumed difference in applying biosecurity measures. For instance, it was anticipated that breeding farms would show better application of biosecurity measures compared to e.g., layer farms because they are higher situated in the poultry pyramid. During the recruitment process, poultry farms were contacted via their veterinary practitioner for participation. Farms received detailed information about the objectives of the study, what was needed from them, what measurements and investigations were planned on the farm (including video camera monitoring), and what the study would provide, e.g., a comprehensive biosecurity assessment of their operation and tailor-made advice to overcome shortcomings. The recruitment process resulted in the acceptance to participate of two broiler, two layer, one duck (there were no more duck farms available at the time of recruitment), and two breeder farms. The remaining 20 farms indicated no willingness to participate. Reasons mentioned for turning down the invitation were as follows: not interested, having a problem with direct (video-camera) observation, no interest to show their biosecurity breaches to us, would cost too much time, and fear of introduction of contamination by researchers. These not-willing-to-participate farms were not different in flock size or structure (private or integrated) compared to the participating farms.

All participating farm owners (n = 7) received a compensation of EUR 500. An intake visit on site was organised to evaluate the baseline quality of on-farm biosecurity using a combination of several scoring tools, including Biocheck.Ugent^TM^ [21], the Dutch AVINED hygiene scan [22], and additional avian-influenza-related risk factor questions. Other methods to identify potential weaknesses were applied simultaneously, such as the use of fluorescent (glowing) gels at doors to identify weaknesses caused by fomites, incoming rain water under doors, and vectors, including vermin, and wildlife-trapping cameras around the farm buildings. The combination of these methods was used to support a farm-specific advice on different types of biosecurity improvements after the end of the study.

Informed consent for data collection (including video camera recording), data management and data storage, and dissemination of results in anonymised form taking into account the European legislation (EU General Data Protection Regulation 2016/679) with respect to protection of privacy [23] was provided in writing through signature by each farm owner.

### 2.2. Hygiene Lock

**Figure 1:**
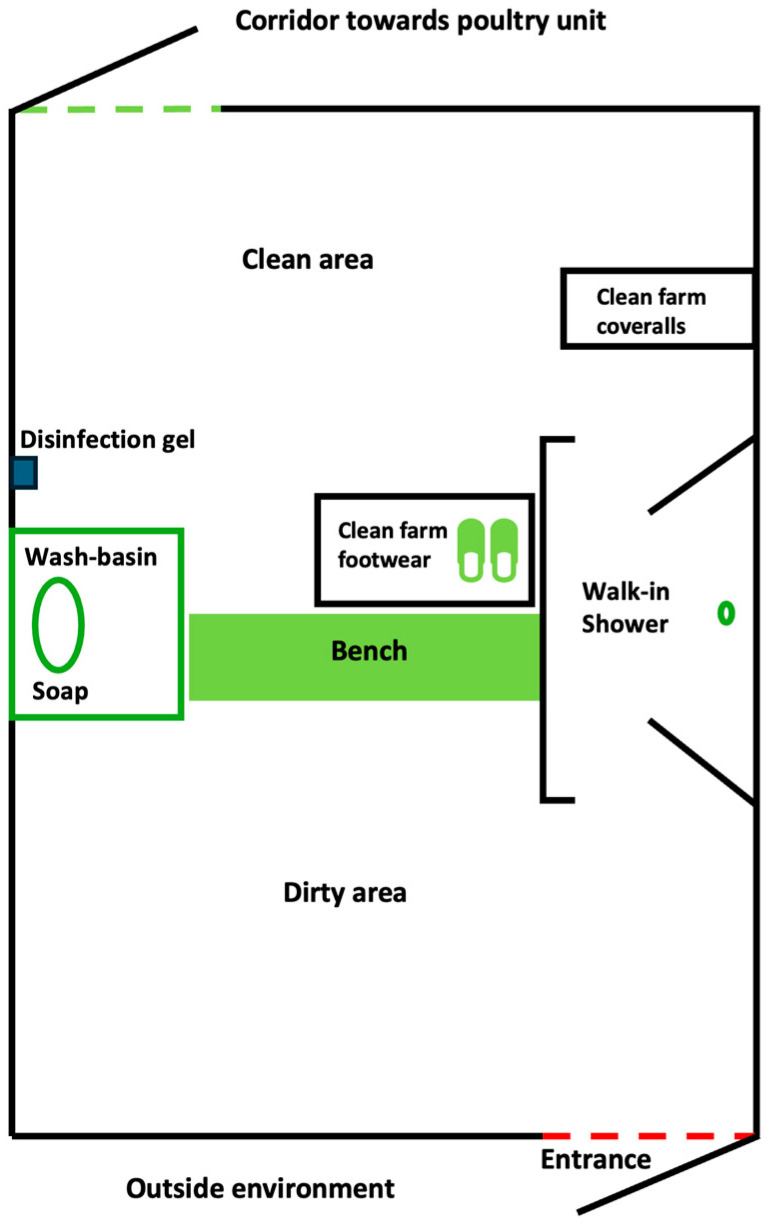
Diagram of an anteroom with a sophisticated hygiene lock design. Separation of a dirty and clean area by a bench. In the dirty area, there is a wash basin where a farmer/visitor would wash hands, leave all external clothes and footwear in a walk-in shower, and shower and put on a clean coverall and clean farm footwear when leaving the walk-in shower.

**Figure 2:**
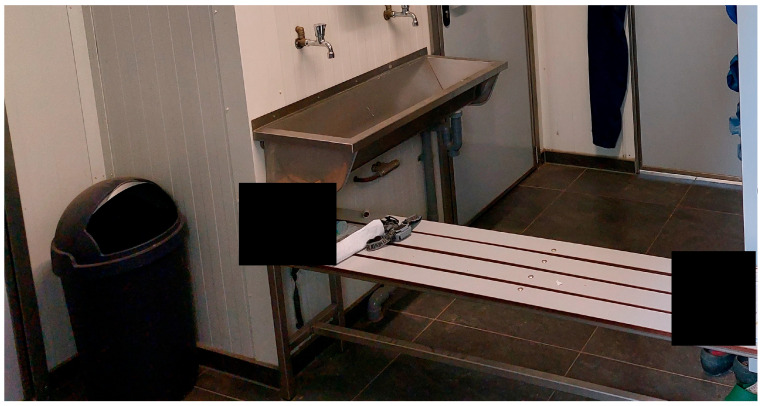
Example of a farm anteroom (taken from the entrance of the poultry building) with a sophisticated hygiene lock design, as applied by some poultry farms in the Netherlands: wash basin to wash hands with soap and disinfection gel dispenser on the wall above the wash basin and paper towel; bench to sit on when changing footwear; on the far right side, a small part of the door towards the walk-in shower is seen.

A hygiene lock is a designated transition area that serves as a biosecurity barrier to prevent introduction of pathogens. It includes a demarcation between two areas that one wants to separate from each other (e.g., a clean and dirty area (Figure 1)) and where one needs to follow hygienic measures, such as changing footwear, washing hands and change into a clean coverall [3,24,25]. In exceptional cases, farms have a farm hygiene lock which is located in a separate building on the premises (private house of the farmer or a special small farm hygiene lock building), where farmer/visitors have to shower and change into farm footwear and farm clothing/coveralls. More commonly, the hygiene lock is located in an anteroom, which is an area between the entrance to the poultry building and the entrance to the poultry unit (or a corridor area after which you would enter the poultry unit), where the poultry is housed. Entrance to the poultry building complex is always via the anteroom. With a sophisticated hygiene lock in the anteroom, a dirty and clean area is separated by a bench. In the dirty area, there is a wash basin where a farmer/visitor would wash hands, leave all external clothes and footwear in a walk-in shower, and shower. The farmer/visitor comes out of the walk-in shower into the clean area, where one changes into clean farm underwear, socks, shirt, coverall, and farm footwear (Figure 1 and Figure 2).

**Figure 3:**
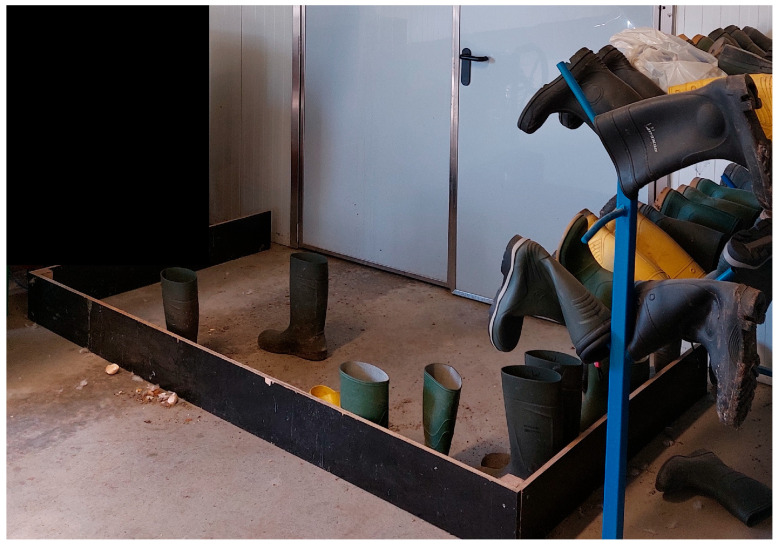
Example of a hygiene barrier where one would change into poultry unit footwear before entering the central corridor that gives access to several different poultry units, as applied by some poultry farms in the Netherlands.

The visitor/farmer would change again in a safe way at a second hygiene barrier to poultry unit footwear when entering the poultry unit where the poultry is housed (Figure 3).

**Figure 4:**
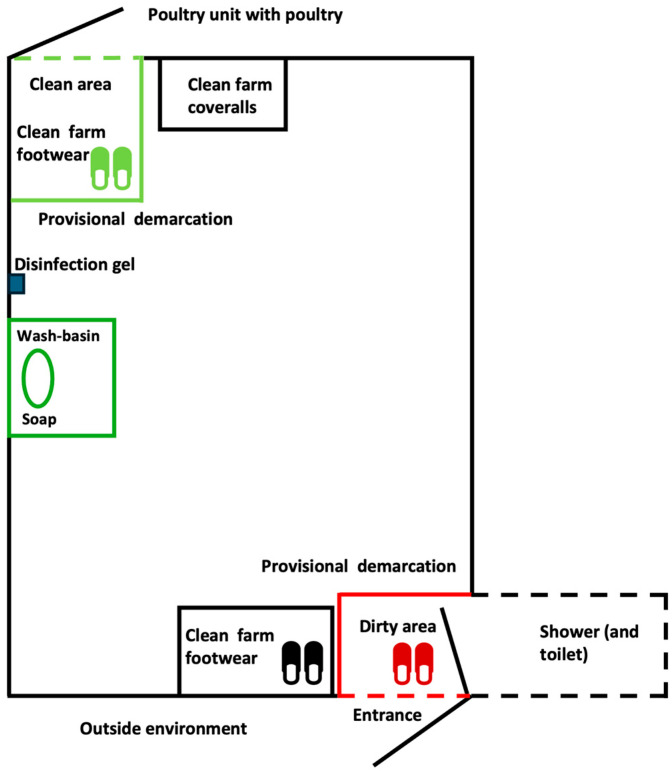
Diagram of an anteroom with a basic hygiene lock design. External footwear is changed for clean farm footwear at a provisional barrier between the dirty outside and clean area just after entering the anteroom. This provisional barrier often consists of small wooden boards with a height of 10–15 cm on the floor for demarcation. If the anteroom is directly connected to a poultry unit housing poultry, the farm footwear from the anteroom is changed for clean poultry unit footwear at another barrier (and putting on a clean coverall) before entering the poultry unit.

**Figure 5:**
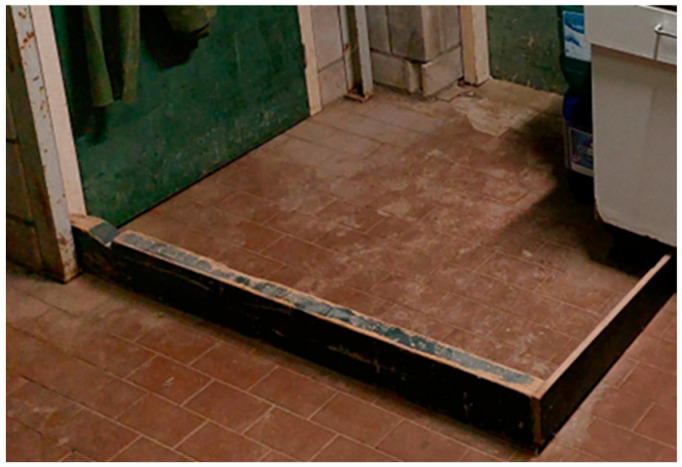
Example of a basic hygiene lock design at the entrance of a poultry building (taken from inside the anteroom towards the entrance door of the poultry building), as applied by many poultry farms in the Netherlands: separation of the dirty and clean area using a wooden board construction; at this point, one would change personal footwear for farm footwear; on the left side, the door towards the shower and toilet.

In a basic hygiene lock design, external footwear is changed for clean farm footwear at a provisional barrier between the dirty and clean area just after entering the anteroom [26]. This provisional barrier consists of small wooden boards with a height of 10–15 cm on the floor for demarcation (Figure 4 and Figure 5).

**Figure 6:**
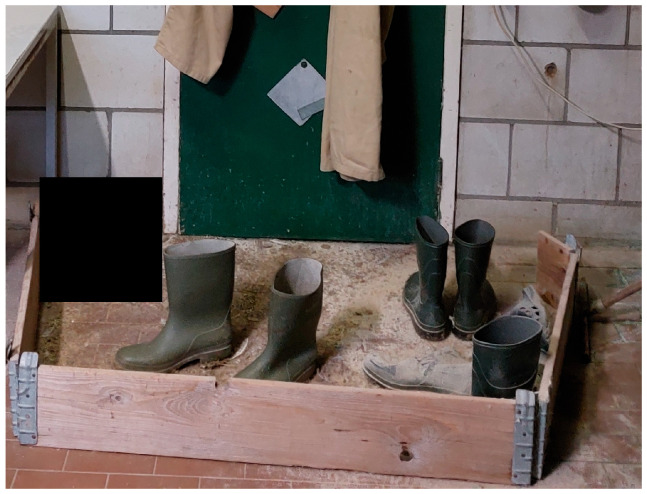
Example of a basic hygiene lock design at the entrance to a poultry unit (taken from inside the anteroom towards the entrance door of the poultry unit), as applied by some poultry farms in the Netherlands: separation of the dirty and clean area using a wooden board construction; at this point, one would change anteroom footwear for poultry unit footwear (and putting on a clean coverall) when entering the poultry unit housing the poultry.

After changing into the clean farm footwear, the visitor/farmer would wash hands with soap and possibly rub the hands with a disinfection gel and put on a clean farm coverall. When entering the poultry unit where the poultry is housed, the farmer/visitor would change again in a safe way to other clean poultry unit footwear (Figure 6).

### 2.3. Study Implementation

The use and analysis of video monitoring data from several weeks of recording per poultry farm is very labour-intensive and, given the small number of farms participating, this study is not meant to make a large number of statistical inferences but to arouse awareness on application of basic biosecurity measures. This is why we selected different poultry production types to gain knowledge possibly linked to production type. Each farm was subjected to the same “intervention”: video cameras were installed in- and outside the poultry building and a laminated poster was attached to the wall inside the anteroom and at doors in the area where video camera monitoring was executed. The text on the poster called for attention that temporary video camera recording was taking place for research purposes.

Video recording was conducted 24 h per-day and 7 days-per-week for a consecutive period of 4 to 6 weeks. This extended recording period was planned in order to capture a possible change in application of biosecurity measures as a consequence of farmers, visitors, and/or transporters being aware of the presence of the video cameras [17]. In order to check this, the proportion of biosecurity breaches at the beginning (first seven days) and at the end of the observation period (last seven days) on a farm was compared.

### 2.4. Description of Study Farms

#### 2.4.1. Farm ID 1

This farm is a breeder farm with a flock size of about 40,000 breeder chickens, divided over several physically separated poultry units. The poultry units are connected to each other by a central corridor situated in front of the units that allows entry to the different poultry units by separate doors. The anteroom is equipped with a toilet, walk-in shower, a bench, and a wash basin to wash hands and to disinfect the hands with a disinfection gel (design like in Figure 1). Visitors have to shower in the walk-in shower in the anteroom and change into footwear and clothing (underwear, socks, T-shirt, and coverall) made available by the farm, whereas the farmer and co-workers change footwear and put on a clean coverall at the bench (not showering). Connected to the anteroom is a separate central room (egg sorting/storage room) where the eggs are sorted and processed into trays; the eggs are supplied to this room from the poultry units via conveyor belts. In this central room, the eggs are stored in trolley racks for a few days before transport to the hatchery (no physical separation between storage and sorting room). From this central room there is a connection (via a door that can be closed) to the central corridor that runs in front of the poultry units, with a provisional barrier (wooden boards construction) where you have to change into clean poultry unit footwear (poultry unit footwear placed within the demarcation; design like in Figure 3). A video camera was placed in the anteroom to cover footwear change, to observe hand hygiene, and putting on a clean coverall. In the central room, a video camera was placed to cover footwear change when entering and leaving the central corridor to the poultry units. The eggs are shipped off from the sorting area via a lockable sliding door to the outside of the building. In this area, a separate video camera was placed to cover movements connected to shipping off eggs.

#### 2.4.2. Farm ID 2

This farm is a layer farm with a flock size of about 50,000 chickens, divided over several physically separated poultry units. These poultry units are connected to an open area (central corridor) where the eggs are sorted and also stored in trolley racks. There is no physically separated room for egg storage. Entrance to the building complex is via a lockable door that is connected to the central corridor. Here, no barrier was present (either a bench or wooden board construction) between the outside of the building and the central corridor. In the central corridor there is wash basin to wash hands, there is a shower room in the central corridor in which visitors have to shower, and the farmer and co-workers do not use the shower (design like in Figure 4, except for absence of a provisional barrier at the entrance to the building). Entrance to the poultry units from the central corridor is via a lockable door and, before this entrance, there is a provisional separation made of a wooden board construction in which you have to change into poultry unit footwear (poultry unit footwear placed within the demarcation; design like in Figure 6). One video camera was placed in the central corridor to investigate change of footwear and hand hygiene on entering the poultry building. Note: this covered the first footwear change from personal footwear to footwear use in the central corridor. At both entrances to the poultry units, video cameras were placed to cover changing footwear. The eggs are shipped off from the central corridor via the door at the entrance to the outside of the building. Egg-shipment movements were also covered by a video camera in the central corridor.

#### 2.4.3. Farm ID 3

This farm is a breeder farm with a flock size of about 40,000 breeder chickens, divided over several different poultry units. Visitors have to shower in a farm hygiene lock, situated in a private separate house of the poultry farmer, about 100 m from the poultry building (farmer and co-worker do not use the shower). Here you can shower and change into farm footwear and farm clothing (underwear, socks, T-shirt, and coverall). Entrance to the building complex is via a lockable door that is connected to a central corridor that connects the poultry units. This entrance is equipped with a provisional hygiene barrier (wooden boards construction) between the outside of the building and the central corridor (design like in Figure 4); here you have to change from the farm footwear into “central corridor” footwear. Before entering the poultry units (via a lockable door) from the central corridor, you have to change into poultry unit footwear. However, there is no (provisional wooden boards) demarcation constructed between the central corridor and the poultry units to physically separate a dirty from a clean area and change footwear accordingly. The boots (poultry unit footwear) are placed in the central corridor near the door to the poultry unit (design like in Figure 6, except for absence of a provisional barrier at the entrance to the poultry units). Connected to the central corridor is an open area where the eggs are sorted and processed into trays. The eggs are supplied to this area from the poultry units via conveyor belts. There is a physically separate egg storage room, connected to the central corridor where the sorted eggs are stored in trolley racks for a few days before transport to the hatchery. The eggs are shipped off from the storage room via a lockable door to the outside of the building. One video camera was placed in this area to cover movements connected to changing footwear and hand hygiene. Two separate video cameras were placed in the central corridor to cover changing footwear when entering poultry units. A video camera was placed in the egg storage room to cover movements connected to shipping off the eggs.

#### 2.4.4. Farm ID 4

This farm is a broiler farm with a flock size of about 70,000 broilers, divided over different poultry buildings. Each poultry building is equipped with an anteroom and basic hygiene lock (design like in Figure 4). Each anteroom was equipped with a separate shower room, toilet, a provisional hygiene barrier with wooden boards when entering the building to change from outdoor into clean farm footwear, a wash basin to wash hands and to disinfect the hands with a disinfection gel, and where one puts on a clean coverall. A second provisional hygiene barrier (wooden boards), where farm footwear was changed to poultry unit footwear (poultry unit footwear placed within the demarcation), was installed just inside the poultry unit when entering the poultry unit from the anteroom via a lockable door (design like in Figure 6). Video cameras were placed in each of the anterooms to cover changing footwear at the first and second hygiene barrier, washing and disinfecting hands, and putting on a coverall.

#### 2.4.5. Farm ID 5

This farm is a broiler farm with a flock size of about 50,000 broilers, divided over different poultry buildings. Each building is equipped with an anteroom and a basic hygiene lock. Each anteroom was equipped with a separate shower room, toilet, a provisional hygiene barrier with wooden boards when entering the building, and a wash basin to wash hands and where one puts on a clean coverall (design like in Figure 4). However, no clean farm footwear is made available by the farm at the first hygiene barrier after entering the building. The farmer and visitors have to take off their personal footwear in the demarcation area, and step with socks into the anteroom. A second provisional hygiene barrier (wooden boards) is installed before the door of the poultry unit where one can put on poultry unit footwear (poultry unit footwear placed within the demarcation) when entering the poultry unit (design like in Figure 6). A video camera was placed in the anteroom of one of the poultry buildings to cover footwear change (entering and leaving the poultry building and the poultry unit), hand hygiene, and putting on a coverall. Furthermore, a video camera was placed outside of the building to cover the introduction of young stock to the poultry unit at the start of the production period.

#### 2.4.6. Farm ID 6

This farm is a layer farm with a flock size of about 25,000 chickens in one poultry unit. At the entrance to the premises, there is a small shed, a farm hygiene lock, in which you have to change into farm footwear and a farm coverall and where you sign your visit into a visitors’ logbook. From this building you have to walk for about 200 m to get to the poultry building. Entrance to the poultry building is via a lockable door that is connected to a central area where the eggs are sorted. There is a first hygiene barrier (wooden boards and a bench) after entering the poultry building, where you have to change farm footwear into “central area” footwear (design like in Figure 4). In the central area, eggs are sorted and processed into trays; the eggs are supplied to this area from the poultry unit via conveyor belts. Between the central egg-sorting area and the poultry unit, there is a central corridor. At the crossing between the central sorting area and the central corridor, there is a provisional hygiene barrier (wooden boards) for a change into poultry unit footwear (poultry unit footwear placed within the demarcation) and putting on a coverall. Entrance to the poultry unit from the central corridor is via a lockable door (design like in Figure 6). Connected to the central egg-sorting area is a physically separated egg-storage room; this room is separated from the central (egg-sorting) area by a sliding door. The eggs are shipped off from the storage room via a lockable door to the outside of the building. A video camera was placed inside the poultry building near the entrance (first hygiene barrier) to cover changing footwear and putting on a coverall. Furthermore, a video camera was placed in the central corridor to cover changing footwear when entering and leaving the poultry unit and a video camera was placed in the egg-storage room to cover egg-shipment movements.

#### 2.4.7. Farm ID 7

This farm is a duck farm with a flock size of about 20,000 ducks. One-day-old ducklings arrive at the farm and are housed in a separate but interconnected building specifically equipped for the early rearing period from a day old, including a heating system. After about 2–3 weeks they are moved to a second (adjacent) building via a covered corridor linking both buildings, where they stay until they are transported to the slaughterhouse. Each building is equipped with an anteroom and hygiene lock (design like in Figure 4). Each anteroom was equipped with a separate shower room, toilet, a first provisional hygiene barrier (wooden boards) when entering the building to change footwear, and a wash basin to wash hands and where one puts on a clean coverall. A second provisional hygiene barrier (wooden boards) was installed just inside the poultry unit when entering the poultry unit from the anteroom via a lockable door (poultry unit footwear placed within the demarcation; design like in Figure 6). In the anteroom of the second building (where the ducks were fattened until transport to the slaughterhouse), a video camera was placed to cover changing footwear (entering and leaving the poultry building and poultry unit), hand hygiene, and putting on a coverall. Furthermore, a video camera was placed outside the poultry building to cover the introduction of young ducklings and bedding material into the poultry unit at the start of the production period.

### 2.5. Definition of Biosecurity Breaches

Desired biosecurity behaviour was based on internationally acknowledged norms published in poultry biosecurity reference guides and handbooks [3,25,27,28]. Although standards and general biosecurity protocols are included in certification schemes and additional measures can be enforced through the “Regulation on veterinary measures for specific animal diseases or Zoonoses” [29] (based on the Animal Act and European Union (EU) Regulation 2016/429 on transmissible animal disease in case of increased risk of avian influenza), the specific biosecurity norms and definitions of breaches used in this study are not laid down in Dutch legislation.

An overview of biosecurity measures that were observed in different areas on the poultry farms with the help of video camera monitoring is shown in Table 1.

The following definitions of biosecurity breaches were used in the different areas on the poultry farm.

#### 2.5.1. Enter/Exit Anteroom from Outside (First Hygiene Barrier)

Desired biosecurity behaviour: changing footwear in the proper way. Breaches: (a) not changing footwear; (b) no footwear available and obligation to move around on socks after passing the first hygiene barrier in the anteroom; (c) not properly changing footwear: stepping out of your personal (outdoor) footwear or farm hygiene lock footwear and touching the floor with your socks (in dirty or anteroom area) before changing into new farm footwear.

#### 2.5.2. Wash/Disinfection of Hands in Anteroom

Desired biosecurity behaviour: washing your hands with water and soap and preferably subsequently rubbing them with disinfection gel upon entering and leaving the anteroom. Breach: not washing your hands with water and soap OR if not washing your hands with water and soap, not rubbing your hands with a disinfectant gel upon entering and leaving the anteroom.

#### 2.5.3. Enter/Exit the Poultry Unit (Second Hygiene Barrier)

Desired biosecurity behaviour: changing footwear in the proper way. Breaches: (a) not changing footwear; (b) not properly changing footwear: stepping out of footwear or, when you do not wear footwear, walking on socks and touching the floor with your socks (in anteroom or clean area poultry unit) before changing into anteroom farm footwear.

Desired biosecurity behaviour: putting on a clean coverall (made available by the farm or bringing a disposable coverall yourself) over your normal clothes in the anteroom before entering the poultry unit where the poultry is housed. Breach: not putting on a coverall before entering the poultry unit.

Desired biosecurity behaviour: disinfecting wheels of trolleys with crates of day-old birds when entering the poultry unit from outside. Breaches: (a) not changing footwear; (b) not disinfecting wheels of the trolleys.

#### 2.5.4. Egg Storage Room

Desired biosecurity behaviour: changing footwear when entering from outside into the egg storage room to ship off eggs; disinfecting wheels of the forklift when entering the egg storage from outside; cleaning and disinfecting the egg storage room after visit by egg transporter. Breaches: (a) not changing footwear; (b) not disinfecting wheels of the forklift or not cleaning or disinfecting the egg storage room after visit by egg transporter.

### 2.6. Video Camera Monitoring

Hikvision low-light Turret 4 Mp video cameras (VC) were used for monitoring, each fitted with a fixed-focus wide-angle 2.8 mm lens (Hikvision, Hangzhou, China; www.hikvision.com). The VCs were positioned on the ceiling of the room (beaming towards the ground) or high on a wall in the room. VCs were connected to a TruVision NVR10 network video recorder with HDMI/VGA video output and a 12 Tb hard disk for storage. Recording of movement of people and material in the critical areas was conducted at 6 frames/s and, under low-light conditions, was facilitated using infra-red LEDs.

### 2.7. Translating Video Recordings into Data for Analysis

Video recordings were replayed on a large LCD monitor. Recordings could be replayed at different speed, and there was a possibility to archive snap-shots of specific video recordings. Event recording is used when frequency is the response dimension of interest [12]. With event recording, initiations of the target behaviour are scored for each occurrence in an observational session. Specified movements of people or material recorded by video camera monitoring were entered into a database: farm identification; farm type; visitor or material identification; date of event; time of start and end of specific event; identification of area within the poultry building; and specified biosecurity breaches observed.

### 2.8. Statistical Analysis

The rate of disregarding biosecurity compliance per movement for each of the above defined breaches was estimated by fitting generalised mixed models with a Poisson distribution [30]. For each defined breach, the daily number of breaches was used as the response variable, the log of the daily number of movements (relevant to the breach) was included as offset, and the farm identifier as random intercept. In addition, these models were used to compare differences in rate of biosecurity breaches between the first week and the last week of the observation period to test the hypothesis of presence of an “observer” effect (Hawthorne effect). A possible “observer” effect could be imagined via the presence of the overt “intervention”, change in biosecurity behaviour because of their knowledge that their actions are being recorded by video camera. Furthermore, differences in the rate of biosecurity breaches between the morning and afternoon of observation days were tested as well as differences between production types.

A pathway analysis of possible biosecurity breaches when entering the poultry building via the anteroom all the way to the poultry unit was executed to quantify the overall probability of compliance. This analysis describes four steps where a breach could be observed: changing footwear (at the first hygiene barrier) when entering the anteroom from outside (possible Breach 1), washing hands with water and soap and/or using disinfection gel in the anteroom (possible Breach 2), changing into a coverall (possible Breach 3), and changing footwear (at the second hygiene barrier) in the anteroom before entering the poultry unit (possible Breach 4). The estimated rates of a biosecurity breach per movement were converted to the Poisson probability of observing a breach per movement and these probabilities were combined in a pathway analysis. The estimated rates of each separate breach were estimated (over all production types) irrespective of earlier breaches in the process from entering the building to entering the poultry unit. The overall probabilities are the product of the probabilities (breach or no breach) for each step in this process (from changing footwear when entering the anteroom from outside at the first hygiene barrier, washing hands, and changing into a coverall to changing footwear when entering the poultry unit at the second hygiene barrier).

## 3. Results

Of all the movements by persons recorded in this study, 59% were linked to the poultry farmer, 28% to co-working family members, 9% to transporters (young stock and egg shipment), and 4% to external visitors (adviser and veterinary practitioner).

There were no indications for a possible Hawthorne (observer) effect; no statistically significant difference (*p* > 0.50) in proportion of biosecurity breaches by persons was observed in the first week compared to the last week of video monitoring on the poultry farms.

The rate of biosecurity breaches by farmer, co-workers, or visitors during daily movements was significantly (*p* = 0.007) higher in the afternoon (>12:00 h) compared to the morning (≤12:00 h).

### 3.1. Entering–Leaving the Anteroom

**Figure 7:**
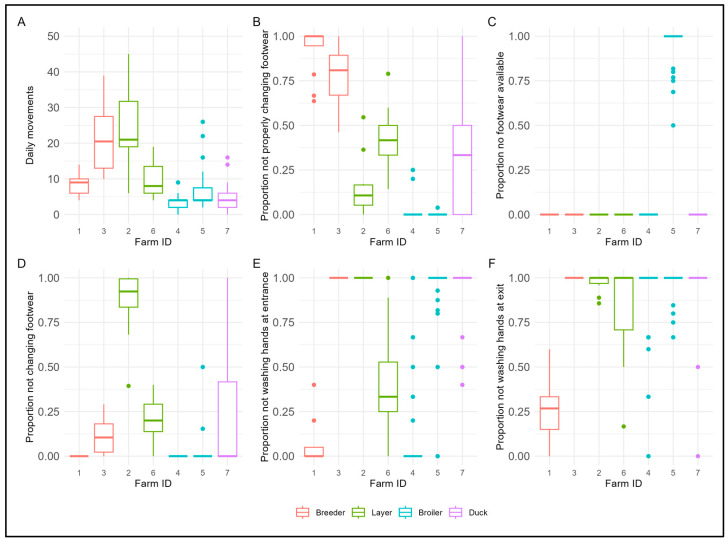
Distribution of daily movements by persons when entering or leaving the anteroom (**A**), proportion of persons not properly changing footwear when entering or leaving the anteroom (**B**), proportion of person movements where no footwear was available in the anteroom to change when entering or leaving the anteroom (**C**), proportion of persons not changing footwear at all when entering or leaving the anteroom (**D**), proportion of persons not washing hands at entering the anteroom (**E**), and proportion of persons not washing hands at leaving the anteroom (**F**), by farm identification and farm type (thick dark line in the box: median; lower end of the box: 25% quantile; higher end of the box: 75% quantile; highest bullet or high end of the vertical line coming out of the box: highest value; lowest bullet or low end of the vertical line coming out of the box: lowest value).

The mean number of daily movements by persons when entering or leaving the anteroom was for the breeder and layer farms at least twice as high compared to those of the broiler and duck farms (Figure 7A). With respect to proper change of footwear at the first hygiene barrier in the anteroom when entering the poultry building, there was one broiler farm (farm ID 5) that had no footwear available to change into, making that a high risk (Figure 7C). On both breeder farms, one of the layer farms, and the duck farm, persons showed a high proportion of not changing footwear in a proper way when entering and leaving the anteroom (Figure 7B). On only one farm (broiler farm ID 4), people showed the highest level of desired proper footwear change when entering or leaving the anteroom (Figure 7B,C). There was a layer farm (farm ID 2) where a high proportion of persons (farmer and co-workers) did not change footwear, with the exception of the visiting veterinary practitioner who used disposable footwear covers (Figure 7D). On four out of seven farms (one layer, one breeder, one broiler, and one duck farm), persons showed a high proportion of not washing their hands with soap or disinfecting the hands when entering the anteroom (Figure 7E). The poultry farmer and family workers on only one breeder farm (Farm ID 1) and one broiler farm (farm ID 4) washed their hands and/or used a disinfection gel consistently after entering the anteroom (Figure 7E). When leaving the anteroom to the outside premises (after visiting the poultry unit), the proportion of persons ignoring hand washing is very high for most of the farms, except for the breeder farm (farm ID 1) (Figure 7F).

In one of the broiler farms (Farm ID 5), mice were observed by video monitoring to be present in the anteroom. Furthermore, this farm fed self-produced wheat to the broilers besides the standard pelleted broiler feed. The wheat was stored in open silos (without a roof cover) situated in a separate shed; separate wildlife camera monitoring installed near the silos showed predominantly sparrows (*Passer domesticus*) feeding on the stored wheat and these birds were able to contaminate the wheat with faecal droppings.

### 3.2. Entering–Leaving the Poultry Unit

**Figure 8:**
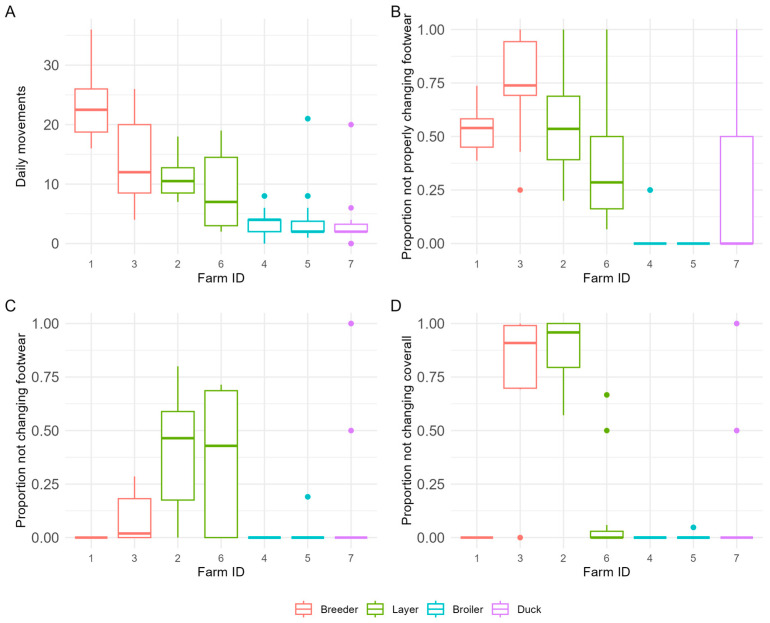
Distribution of daily movements when entering or leaving the poultry unit (where poultry is housed) (**A**), proportion of persons not properly changing footwear when entering or leaving the poultry unit (**B**), proportion of persons not changing footwear when entering or leaving the poultry unit (**C**), proportion of persons not changing in and out of a clean coverall when entering or leaving the poultry unit (**D**), by farm identification and farm type (thick dark line in the box: median; lower end of the box: 25% quantile; higher end of the box: 75% quantile; highest bullet or high end of the vertical line coming out of the box: highest value; lowest bullet or low end of the vertical line coming out of the box: lowest value).

The mean number of daily movements of persons entering or leaving the poultry unit was for the breeder and layer farms at least twice as high compared to those of the broiler and duck farms (Figure 8A). With respect to properly changing footwear at this second barrier, the highest level of compliance was observed for both broiler farms (Figure 8B). Most of the poultry farmers, co-workers, and visitors showed a high level of compliance with respect to putting on a poultry unit coverall before entering the poultry unit, except for one of the breeder and one of the layer farms (Figure 8D).

### 3.3. Entering–Leaving the Egg Storage Room

When storing eggs in the egg storage room by the poultry farmer and/or family co-workers or when eggs were shipped off the farm by a transporter, no footwear change or disinfecting footwear was observed in 100% of the cases. Wheels of the forklift or wheels of the trolleys with egg trays were not disinfected when entering the egg storage room (100% breaching). Furthermore, during the observation period on each egg-producing farm, the egg storage room or area where they are stored was not cleaned and disinfected by the farmer after each shipping of eggs off the farm.

### 3.4. Introduction of Young Stock into Poultry Unit

When young stock (duck and broiler farm) was delivered to start a new production round, the poultry farmer, (family) co-workers, and transporters showed no compliance at all with respect to changing or possibly disinfecting footwear. Trolleys with trays with young stock entered the poultry unit without disinfection of the wheels. At the broiler farm, the concrete floor outside the poultry unit was sprayed in the morning before arrival of the young stock by transporter, but it may be questioned if the level of disinfection was high enough and the floor had dried up when the transporter arrived, which considerably diminishes disinfection properties.

### 3.5. Introduction and Refreshment of Bedding Material into Poultry Unit

When bedding material (duck farm) was brought into the poultry unit before the start of the production period (and additional bedding material during the production period when ducks were present in the poultry unit), the poultry farmer showed a low level of compliance with respect to changing or disinfecting footwear (100% breaching). Furthermore, bedding material coming from a storage house was stored for a short period on the premises close to the large sliding door entrance of the poultry unit before it was entered into the poultry unit. This might have contaminated the bedding material while in contact with concrete floor of the farm yard. In addition, the wheels of the forklift bringing the bedding material into the poultry unit were not disinfected before entering the poultry unit.

### 3.6. Pathway Analysis of Biosecurity Breaches

**Figure 9:**
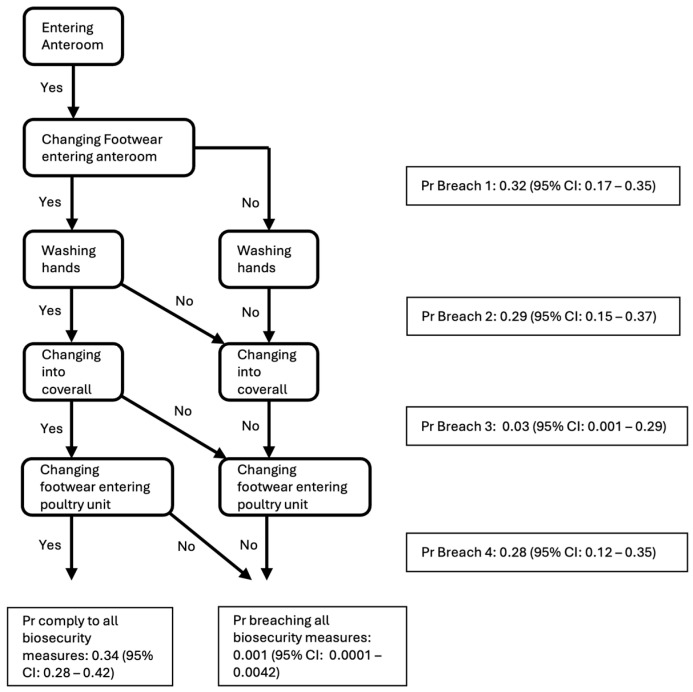
Pathway analysis of biosecurity breaches (using calculated breaching probabilities) when entering the anteroom from outside concerning changing footwear (Breach 1), washing hands with water and soap and/or using disinfection lotion on the hands in the anteroom (Breach 2), changing into a coverall (Breach 3), and changing footwear in the anteroom before entering the poultry unit (Breach 4).

Estimated mean probability of Breach 1 was 0.32 (95% confidence interval (CI): 0.17–0.35) per movement; 0.29 (95% CI: 0.15–0.37) per movement for Breach 2; 0.03 (95% CI: 0.001–0.29) per movement for Breach 3; and 0.28 (95% CI: 0.12–0.35) per movement for Breach 4 (Figure 9). Proper changing of footwear and good hand hygiene showed the highest breaching probability, while putting on a coverall showed the lowest breaching probability. The estimated mean probability of lack of compliance for all four potential breaches when entering the building from outside all the way to the poultry unit was very small: 0.001 (95% CI: 0.0001–0.0042) (product of probabilities committing to all four breaches). However, the probability that they comply with all the biosecurity measures in these four steps was modest: 0.34 (95% CI: 0.28–0.42).

## 4. Discussion

The pathway analysis indicated that the estimated mean probability of lack of compliance for all four potential breaches by people entering the building from outside all the way to the poultry unit was very low but that the probability that they comply to all the biosecurity measures in these four steps was also poor. Proper changing of footwear and good hand hygiene showed the highest breaching probability, while putting on a coverall showed the lowest breaching probability across study farms. The implication of this finding is difficult to foresee. Unfortunately, we do not know the relative importance or impact of these biosecurity breaches because we lack insight into the transmission probabilities of a pathogen when compliance to a specific biosecurity measure is breached. The combination of the probability of a compliance breach with the transmission probability of that breach would yield the true impact of such a biosecurity breach. Therefore, there is a need for future research to estimate transmission probabilities of specific biosecurity breaches, for instance, via environmental sampling (e.g., environment outside the poultry buildings, footwear, and hands, coverall).

The good news is that it is actually possible to have a high level of biosecurity compliance in practice, as shown by one broiler farm in our study. But, at the same time, our study results show that there is still serious work to be done; a low level of biosecurity compliance by farmers, assisting family members, and farm personnel in the anteroom, especially with respect to footwear change and hand hygiene, was observed in six out of the total seven participating farms. Furthermore, a low level of biosecurity compliance was observed by external transporters, especially during egg loading on egg-producing farms and placement of young stock inside the poultry unit (broilers and ducks). Transport companies should work according to prescribed procedures, and auditing should help to keep them on track. But it would also help if poultry farmers take responsibility and clean and disinfect, e.g., egg storage rooms every time eggs are shipped off the farm by a transporter. Another important finding is that the presumed higher degree of biosecurity compliance of breeders compared to other production types participating in this study is not confirmed by the data.

It should be noted that selection bias might be hampering the interpretation of the results. A relative high proportion of poultry farms contacted for participation in the study declined. Farms that were willing to participate might systematically differ from farms that refused participation. All except for one of the participating poultry farms showed a high degree of lack of biosecurity compliance. It should be noted that we worked with poultry farmers that experienced one or more HPAI outbreaks on their farms and it would be logical to assume that, because of that experience, they had a higher level of biosecurity awareness (bias) [26]. This could suggest that the lack of biosecurity compliance across our target population may be even higher than perceived in this study. On the other hand, there were farms that declined on the basis that they would not allow non-essential visitors to the farm to avoid risks of introduction of pathogens from outsiders. However, one should keep in mind that the focus of these farmers on threats coming from visitors does not implicitly imply that they are good biosecurity compliers on their own farms.

The use and analysis of video-monitoring data from several weeks of recording per poultry farm is very time consuming. More farms participating in the study would increase the precision and representativeness but, for practical and financial reasons, one has to limit the number of farms in the study. It is therefore not possible to indicate if the study results are representative for the total Dutch poultry sector. But the observed low level of biosecurity compliance by farmers, assisting family members, farm personnel, and transporters in this study should be a wake-up call. Especially considering the high risks of introduction of HPAIv as observed in the Netherlands in recent decades [31,32], the required level of biosecurity and consistent compliance by farmers, co-workers, and all farm visitors should be very high and leaves little to no room for mistakes.

The poultry farmer, family co-workers, personnel, and visitors can potentially cause the highest biosecurity risk to any poultry farm due to their frequent movement around poultry units, inherent management tasks, and potential ignorance, indifference and/or carelessness being therefore the most likely cause of disease introduction and spread [33]. People can carry contaminations on footwear, clothes, and on their hands [25], and, when entering poultry buildings, measures must be taken to decrease the likelihood of accidentally introducing pathogens by complying to stringent biosecurity measures. Our study indicates that the proportion of biosecurity breaches during daily movements was significantly higher in the afternoon compared to the morning. It is speculated that this might be caused by fatigue having an effect on alertness and awareness. Another possible reason might be that this is related to different types of activities during the day.

### 4.1. Hand Washing

By manipulating equipment, bedding material, feed, and executing tasks outside on the premises, hands of a poultry farmer are exposed to an array of microorganisms. In order to decrease the risk of transporting pathogens into the poultry unit by contaminated hands, proper hand cleaning is essential. Under farming conditions, hands are mostly visibly soiled and it is recommended to wash hands with water and soap, rinse, and especially dry the hands afterwards. When not drying the hands, residual moisture creates an interface that allows movement of microorganisms from hands to contact surfaces [24]. Soaps are products that are based on detergent and contain esterified fatty acids and sodium or potassium hydroxide. The cleaning activity of soap takes away dirt, soil, and various organic substances from the hands [34]. Soaps have no to minimal antimicrobial activity but washing hands with soap is able to remove transient flora that is lightly attached to the hands [35]. The antimicrobial activity of alcohol-based hand antiseptics can be attributed to their ability to denature proteins [35]. Using a disinfectant after washing hands will therefore further decrease risk of transportation of contaminants.

A field study by van de Giessen et al. [36] indicated that washing hands before tending broiler flocks was correlated with a reduced risk of Campylobacter infections in broiler flocks. A Canadian study using video-camera surveillance found not washing hands at the entrance of the poultry unit in 79% of visits requiring hand washing and not washing hands at exit in 76% of visits requiring hand washing [4]. A farmer-reported hand-washing study (which might produce biased results) in the USA and Thailand indicated that hands were never or sometimes washed in 25% (Minnesota study), 40% (Wisconsin study), and 65% (Thailand study) of events after poultry was contacted [37]. Dorea et al. [38] found self-reported absence of washing hands by farmers in US broiler farms to be 10–13% and 15–20% for visitors, with the notion that self-reported results might be biased (underestimation). A study on French duck farms showed hand washing not to be possible or unlikely to be effective in more than 70% of farms [39]. Van Staaveren et al. [40] showed hand washing to be executed before entering the sheds in only 44% (farmer-reported) of Canadian turkey farms. These earlier investigations confirm a comparable level of disregard for hand washing as observed in our study (29%, 95% CI: 15–37%) and the need for attention to upgrade this biosecurity measure on poultry farms.

### 4.2. Changing or Disinfecting Footwear

When farm workers changed footwear between poultry units on the same premises, the odds of an HPAI outbreak on commercial layer farms decreased by a factor 6 in a case–control field setting in South Korea [7]. Proper boot-dip disinfectant use [41] and use of separate boots between visits of broiler houses [36] was associated with a decreased risk of Campylobacter infections in broiler flocks. These studies emphasise footwear change or proper disinfection of footwear to be an important biosecurity tool. An assessment of farm-level biosecurity in the UK by Knight-Jones et al. [42] indicated that poultry farmers changed footwear before entering the poultry unit in only 14% of cases, and this slightly increased to 23% after having experienced an HPAI outbreak on the farm. Farmer-reported practice of changing footwear (sometimes or always) when entering the poultry unit by farmers was only 35% (Minnesota study), 25% (Wisconsin study), and 0% (Thailand study) [37]. Farmer-reported practice of never changing footwear by the poultry farmers themselves when entering the poultry unit was 42 to 54% in two different areas of the US, while this was 4 to 5% for visitors [38]. Greening et al. [43] indicated a farmer-reported practice of always or often using foot baths by less than 60% of New Zealand commercial poultry farms. Delpont et al. [39] reported changing of footwear by the farmer and visitors to be executed in only 20% and 33% of French duck farms, respectively. Our study results show a higher level of proper footwear change compliance, especially when entering the poultry unit (which is the last and most important hygiene barrier), compared to the above-mentioned studies.

### 4.3. Introduction of Young Stock into Poultry Unit

Any equipment brought into the poultry unit while there are poultry present or at the start of a production round when the unit is cleaned and disinfected should be disinfected before entry [33]. At the start of a production round on a broiler or duck farm, one-day-old birds are transported to the farm by a hatchery. One-day-old broiler chicks and ducklings are transported in crates, and crates are stored in moveable trolleys. It is common practice to unload the trolleys with chicks and ducklings from the transport car and roll them over the pavement into the poultry unit and unload the young animals then in the unit. Hygiene protocols for visiting poultry farms [44] indicate that the external transporter is prohibited to enter the poultry unit together with the trolleys. Our study indicates that transporters, despite the instructions in the visitor protocol, sometimes enter the poultry unit, helping the farmer to get trolleys inside the poultry unit; and, when they enter the poultry unit with the trolleys, they do not change footwear or disinfect footwear, creating a risky situation. Furthermore, it occurs that farmers help pushing the trolleys from the transport car into the poultry unit and, while doing that, do not change footwear or disinfect footwear, risking introducing contamination into the poultry unit.

### 4.4. Egg Transport

Hygiene protocols for shipping off eggs from egg-producing poultry farms and supply of trays, pallets, and roll-containers to the poultry farm [45] indicate that the external transporter has to change into a disposable coverall, wear clean boots and disposable over-boots, and that the transporter is only allowed into the egg-storage room, not in the unit where poultry is housed. Unfortunately, a considerable proportion of egg-producing farms have no separate egg-storage room, so, inherently, this creates a biosecurity problem because you have crossing of (possibly contaminated) pathways in one common room where (a) eggs are coming to and are processed and packed, supplied by conveyor belts connected to the unit where the poultry is housed; (b) eggs are stored to be shipped off by an (external) transporter; and (c) there is a connection to the units where the poultry is housed. When the eggs are stored in a separate storage room (much better starting situation), you still can have a biosecurity problem, as shown by our study, when the storage room is not cleaned and disinfected by the farmer after eggs are shipped off by an external transporter because you have crossing of pathways of the farmer bringing eggs to the storage room and moving around in the common room connected to it as described above.

### 4.5. Determining Biosecurity Breaches

It is quite demanding to properly assess breaches of biosecurity, as day-to-day application of biosecurity measures cannot be correctly judged, unless video camera or electronic tracking and tracing devices are installed on farms [46,47]. So far, these instruments are only used in research situations on a small number of farms. As a simpler and less demanding alternative, biosecurity surveys have been developed over several decades in the past [21,48,49,50]. They typically consist of a list of questions (yes/no answers) to determine whether a preventive measure is available or would be applied (self-reported). The hygiene check of the Dutch poultry industry [22] uses a comparable procedure. The weak point with these questionnaires is that respondents may be motivated to give the answer that is most desirable, regardless of whether that answer is accurate [48]. More importantly, it has nothing to do with how poultry farmers actually apply biosecurity measures on a day-to-day basis. Our study results obtained by direct observation endorse the notion that these questionnaires have strong limitations in actually assessing the level of biosecurity and, in particular, day-to-day application.

### 4.6. Video-Camera Monitoring in a Research Setting

Insight into the actual application of biosecurity measures by poultry farmers in a research setting can be obtained by direct observation. Direct observation of participants in observational research may improve compliance to biosecurity measures unintentionally as a consequence of the awareness of being observed [15]. But the use of video cameras instead of human observers may limit the observer effect [16]. A possible observer effect may be reduced with time, i.e., habituation may take place during a long observation period [19]. That was the reason why an observation period of four or more weeks was used in this study. Comparison of the rate of biosecurity breaches at the beginning and at the end of the study period in our study revealed no indications for presence of an observer effect and supported the belief that the study method with the video cameras produces valid results. Furthermore, these results may be an indication that, in the future, the observation period for comparable investigations with video-camera monitoring on other poultry farms in the Netherlands may be shortened without losing out on validity, and this saves research budget. It should be mentioned that the use of video-camera monitoring raises privacy concerns: concern about being monitored; fears that the results derived from electronic compliance monitoring systems could subsequently be used in a pejorative manner; concerns about punishment, dismissal, or negative performance review; and concerns about showing video footage to third parties [16]. Therefore, the use of video cameras in observational studies requires extensive preparation, information, and communication resulting in a clear and legally sound informed consent from the participants.

### 4.7. Technologies to Monitor Biosecurity Compliance

It is clear that monitoring the application of biosecurity measures is needed to uphold and sustain compliance. Racicot et al. [47] showed that real-time feedback of monitoring results helps improve biosecurity compliance. The use of new technologies, like radio-frequency-identification-based (RFID) real-time continuous automated monitoring systems are first steps to provide for that need, but these developments are still in their infancy [47]. A present-day and down-to-earth monitoring solution would be to use relatively cheap wildlife video cameras by the veterinary practitioner in the framework of his herd-health supervision program. Veterinary practitioners are a primary source of information and expertise and play a pivotal role in influencing farmer behaviour [51,52]. These cameras can be installed temporarily for a couple of days in the anteroom, egg storage, and near poultry building and poultry unit entrances. Video analysis results are discussed with the farmer to detect flaws in biosecurity behaviour of the farmer, co-workers, and visitors and steps for improvement are discussed.

### 4.8. Future Work

This study indicates the presence of a low level of biosecurity compliance in a study population of Dutch poultry farms with a recent history of HPAIv infection. When sharing these observations with a selection of Dutch specialised poultry practitioners for feedback, they indicate that these biosecurity shortcomings are a common issue on much more poultry farms in the Netherlands and not only on farms with a history of HPAIv infection. It should be noted that this is not a unique phenomenon strictly linked to the Dutch poultry industry. When comparing the findings of this study with reports and research studies from other countries, it is clear that a lack of biosecurity compliance on poultry farms is a common issue all over the world (see above in the Section 4.1 and Section 4.2). Therefore, there is a clear need for an engaging awareness and training campaign for poultry farmers and their co-workers as well as for transporters to promote good practices in biosecurity compliance. Important reasons why farmers do not comply with biosecurity measures on their farms are, among others, insufficient knowledge of biosecurity issues and low awareness of benefits [50,53,54]. Farmers have often longstanding biosecurity practices that are resistant to behaviour change plans [55] and there is agreement in the literature that levels of uptake of biosecurity measures on livestock farms are low [52]. To design and implement a coaching program, taking into account differences between poultry production types (layers, breeders, broilers, ducks, and turkeys), to change biosecurity behaviour of poultry farmers will be a large endeavour that will take a long breath.

## 5. Conclusions

- Substantial biosecurity breaches were observed on the majority of poultry farms in our study when farmers, co-workers, and transporters entered/left the anteroom and/or the poultry unit or introduced young stock or bedding material to the poultry unit.
- There was an example of a poultry farm that showed good biosecurity behaviour so, in practice, it is actually possible.
- There is a need for future research to estimate transmission probabilities of specific biosecurity breaches in order to understand the level of risk they might produce.
- There is a need for an engaging awareness and training campaign for poultry farmers and their co-workers as well as for transporters to promote good biosecurity practices.

## Figures and Tables

**Table 1:** Overview of biosecurity measures that were observed in different areas of the poultry farm (and the desired biosecurity behaviour) with the help of video camera monitoring.

Area on Poultry Farm	Biosecurity Measure	Desired Biosecurity Behaviour
Anteroom	Changing footwear Washing/disinfecting hands	Properly changing footwear Properly washing hands
Poultry unit	Changing footwear Coverall	Properly changing footwear Using a clean coverall Disinfecting trolley/forklift wheels when young animals or bedding material is brought in
Egg storage room	Changing footwear	Properly changing footwear Disinfecting forklift wheels or cleaning and disinfecting room after visit by egg transporter

## Data Availability

The raw data supporting the conclusions of this article might be made available by the authors on reasonable request.
